# Supplementary material for: Trends in Fomepizole Use for Acetaminophen Poisoning in the United States; 2013–2024
Source: J Med Toxicol. 2025 Jul 31;21(4):404–8. doi: 10.1007/s13181-025-01091-8 (PMC12511483; doi:10.1007/s13181-025-01091-8)
Supplement: Supplementary file 2 — Supplementary Material 2 [file 13181_2025_1091_MOESM2_ESM.docx]

Appendix B.

Table 1. Raw counts of cases and outcomes by year

|  | 2013 (N=2,056^a^) | 2014 (N=2,541) | 2015 (N=2,927) | 2016 (N=3,519) | 2017 (N=4,421) | 2018 (N=5,460) | 2019 (N=5,888) | 2020 (N=6,192) | 2021 (N=7,685) | 2022 (N=7,679) | 2023 (N=7,560) | 2024 (N=8,002) |
| --- | --- | --- | --- | --- | --- | --- | --- | --- | --- | --- | --- | --- |
| Outcome | N (%) | | | | | | | | | | | |
| Fomepizole | <10^b^ | <10 | <10 | 15 (<1%) | 18 (<1%) | 16 (<1%) | 33 (<1%) | 59 (<1%) | 170 (2%) | 286 (4%) | 377 (5%) | 502 (6%) |
| Death | 44 (2%) | 70 (3%) | 62 (2%) | 103 (3%) | 109 (2%) | 136 (2%) | 156 (3%) | 188 (3%) | 234 (3%) | 235 (3%) | 234 (3%) | 233 (3%) |
| Liver Trans. | <10 | <10 | <10 | <10 | <10 | <10 | <10 | <10 | 12 (<1%) | <10 | 14 (<1%) | <10 |

Note: ^a^N represents number of hospitalized patients who received NAC. ^b^Count values below 10 cannot be explicitly provided due to Epic Cosmos system anonymity requirements.

Table 2. Logistic regression results predicting liver transplantation

|  |  | Liver Transplant | |
| --- | --- | --- | --- |
| Predictor |  | OR (95% CI) | P |
| Fomepizole and NAC (ref: NAC alone) |  | 6.15 (2.61, 14.47) | < .001 |
| Sex (ref: Female) |  | 90.09 (0.49, 1.64) | .733 |
| Ethnicity (ref: Hispanic) |  | 1.03 (0.41, 2.59) | .952 |
| Race (ref: White) |  |  |  |
| American Indian & Alaska Native |  | <0.01 (<0.01, 9.6e^97^) | .931 |
| Asian |  | 0.32 (0.03, 3.60) | .354 |
| Black |  | 0.37 (0.07, 1.87) | .231 |
| Native Hawaiian or Pacific Islander |  | <0.01 (< .01, inf) | .999 |
| Other |  | 1.11 (0.21, 5.89) | .903 |
| Age |  | 1.01 (0.99, 1.02) | .443 |
| SVI |  | 2.21 (0.84, 5.82) | .109 |

SVI: Social vulnerability index
